# Validation of ‘POIBA-How do we eat?’ questionnaire in 9-10 years old schoolchildren

**DOI:** 10.1080/16546628.2017.1391665

**Published:** 2017-10-30

**Authors:** Carles Ariza, Teresa Arechavala, Sara Valmayor, Gemma Serral, Albert Moncada, Luis Rajmil, Anna Schiaffino, Francesca Sánchez-Martínez

**Affiliations:** ^a^ Agència de Salut Pública de Barcelona (Public Health Agency of Barcelona), Barcelona, Spain; ^b^ Ciber de Epidemiología y Salud Pública (CIBERESP), Madrid, Spain; ^c^ Institut d’Investigació Biomédica Sant Pau (IIB Sant Pau), Barcelona, Spain; ^d^ Ajuntament de Terrassa, Terrassa, Spain; ^e^ Agència de Qualitat i Avaluació Sanitàries de Catalunya (AQuAS), Generalitat de Catalunya, Barcelona, Spain; ^f^ IMIM-Institut de Recerca Hospital del Mar, Barcelona, Spain; ^g^ Hospitalet de Llobregat, Institut Català d’Oncologia, Spain

**Keywords:** Food frequency questionnaire, children, 24-hour food recall, validation

## Abstract

**Background**: It is difficult to obtain good food reports with Food Frequency Questionnaires (FFQ) among children. In addition, validated questionnaires are scarce.

**Objective**: The aim of this study was to validate the ‘POIBA-How do we eat?’ (POIBA-HDWE) FFQ and whether it could be administered to children under 10 years of age.

**Design**: We validated the FFQ POIBA-HDWE as part of the Childhood Obesity Prevention Program (POIBA project) in Barcelona. Forty-two out of 63 primary school students (9–10 years old) answered three questionnaires: FFQ POBA-HDWE; another questionnaire, ‘POIBA-How do our children eat?’ (POIBA-HDOCE), which was administered to the children’s parents; and the 24-h recall computer program ‘Young Adolescents’ Nutrition Assessment on Computer’ (YANA-C), which was used on three different days as a gold standard. We tested for correlations using the Spearman test for non-parametric variables.

**Results**: We found low compliance with food recommendations (<50%). The POIBA-HDWE and POIBA-HDOCE questionnaires showed a moderate correlation for soft drinks (r = 0.49; *p* < 0.01), nuts (r = 0.59; *p* < 0.01), dairy products (r = 0.41; *p* < 0.01) and juices (r = 0.49; *p* < 0.01). There were moderate correlations between POIBA-HDWE and YANA-C for fried potatoes (r = 0.42; *p* = 0.01), dairy products (r = 0.53; *p* < 0.01), juices (r = 0.41; *p* < 0.01), and grains(r = 0.50; *p* < 0.01). Food frequency questions showed a homogeneity of 0.69, and a sensitivity of over 60% for all food items except chips (37.5%) and sweets (51.7%).

**Conclusions**: The POIBA-HDWE FFQ showed moderate correlations with the gold standard, high sensitivity for most food types and acceptable internal consistency. It is an easy and affordable tool for recording food frequency in children under 10 years old.

## Introduction

Excess weight, which includes both overweight and obesity, has been a widely studied risk factor due to its association with various ailments such as cardiovascular disease. In 2012, the prevalence of overweight and obesity among children aged 8–13 years in Spain was 30.7% and 14.7%, respectively [1], which raised concern because excess weight is predictive of health problems in adulthood [2,3].

Eating habits can be assessed using various tools, such as dietary recalls and food diaries, etc. Food frequency questionnaires (FFQ) are widely used for their cost-effectiveness and flexibility, although the child population is a difficult target because of the difficulty in obtaining good food reports. Parents can be used as a proxy if children eat with them at home, but most primary school pupils eat at school and reliable information is obtained through the children’s self-reports [4].

Children’s age determines their ability to complete a FFQ since memory development is not complete. It is well known that seven-year-olds are able to provide information on their eating habits [4–6]. Furthermore, FFQ validation studies administered to children under 10 years old have shown moderate correlation compared with the reference method. FFQs can be administered to children, but must be easily understood by the age group studied. Some authors question the capacity of FFQ as an instrument for evaluating the relationship between disease and absolute intake of energy or protein [7]. However, the FFQ is still considered a good tool for studying the evolution of food intake in the field of health promotion [5].

Validating FFQs in dietary studies should be a priority [8]. Several gold standards have been described, such as direct observation, doubly-labelled water or 24-h recalls [8–11]. An example of the latter is the software ‘Young Adolescents’ Nutrition Assessment on Computer’ (YANA-C), which allows children to log the food groups and portions they ate the previous day. This software has been further developed and validated for use in various European contexts, and can also be used as a gold standard [12,13].

Methods for recording food intake in children under 10 years old are scarce and unavailable in our context [8]. In Barcelona, we designed ‘POIBA-Cómo comemos?’ (POIBA-How do we eat? [POIBA-HDWE]) FFQ in the context of the childhood obesity prevention programme ‘Prevención de la Obesidad Infantil en Barcelona’ (Prevention of Infant Obesity in Barcelona – POIBA Project). The aim of this study was to validate the POIBA-HDWE FFQ using the YANA-C software tool as the gold standard, and to determine whether the questionnaire could be administered to children under 10 years of age.

## Methods

### Study design and sample

**Figure 1.**
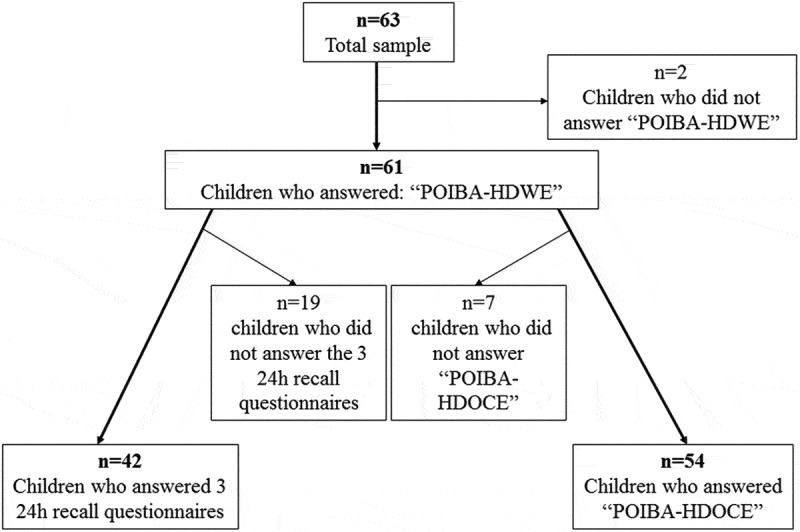
Flowchart of completion of the three study tools by the sample.

This is a validation study of the POIBA-HDWE FFQ designed as part of the POIBA project in Barcelona. The study population was a convenience sample of 63 students aged 9–10 years from two different subsidized primary schools. Sixty-one students completed the POIBA-HDWE questionnaire in 2012 (Figure1); we excluded two students who did not complete the questionnaire. Of these 61 students, 42 completed the 24-h food recall YANA-C three times. Fifty-four parents of the 61 pupils completed the ‘POIBA-How do our children eat?’ (POIBA-HDOCE) questionnaire.

### Procedure

We designed two different questionnaires to assess the children’s normal food intake: POIBA-HDWE and POIBA-HDOCE, and used the 24-h recall computer program YANA-C as the gold standard [12].

POIBA-HDWE is a self-administered questionnaire used in the POIBA project, directed at children under 10 years old. It is based on four different food questionnaires: the Enkid FFQ [14], designed for the EnKid study in Spain, the FFQ designed by the Spanish Pharmacy Association and administered to children aged 11–13 years [15], the Perseo FFQ designed by the Spanish Agency of Consumption, Food Safety and Nutrition (AECOSAN) [16], and finally, the FFQ designed in the CATCH study and administered to children aged 10–12 years in Texas (USA) [17]. Some survey questions had been validated previously, while others that were useful for different variables of our project have not been validated.

We conducted a pilot study to evaluate the intelligibility of the questionnaire for children aged 9–10 years and modified it with pictures to illustrate the consumption frequency gradient. The resulting questionnaire contained 74 questions, including items on socio-demographic characteristics, food preferences, general nutritional knowledge and regular food frequency consumption. There were 20 food intake questions, and all were related to consumption over the previous four weeks. Response options were different between food groups, and categories range from ‘less than once per month’, ‘1–3 times per month’, ‘1–3 times a week’, ‘4–6 times a week’, ‘once a day’, ‘≥2 times a day’, and ‘≥3 times a day’. We assessed intake of bread, potatoes (fried and not fried), rice, pasta, grains, vegetables, fruits, juices, dairy products, legumes, meat, fish, eggs, nuts, soft drinks, chips, milk-based desserts, pastries, sweets and cold meat. The questionnaire included information about age, socioeconomic status (Family Affluence Scale-FAS [18]), weight (kg) and height (cm) measured objectively by staff from the Public Health Agency of Barcelona. The POIBA-HDWE FFQ can be accessed at: http://www.aspb.cat/poiba/

Since parents can be a proxy for obtaining children’s food consumption, we designed a second FFQ to be completed by parents, POIBA-HDOCE. It included 48 questions on the same food items and categories as the POIBA-HDWE, but we grouped pasta, rice and grains together in one question, and did not differentiate between fried potatoes and other forms of potatoes.

YANA-C is a European software program for children aged 11–13 years, and was designed to record their food intake during the previous 24 h. It is a self-administered 24-h recall tool that includes information from six meals: breakfast, mid-morning snack, lunch, mid-afternoon snack, dinner, and evening snack, and can be used as a criterion standard for FFQ validation. The questionnaire must be administrated to each child at least three times on three different days (including Monday to obtain a representation of a Sunday meal), and in different weeks in order to record average food intake for a whole week [12,13].

POIBA-HDWE was administered during school hours and the students filled it out directly on the computer, spending less than one hour on each questionnaire. The teacher of each class was present to resolve any questions. Regarding YANA-C administration, an expert was present to guide the children in understanding the material and answering the questions appropriately. Parents received the POIBA-HDOCE questionnaire during a meeting at the beginning of the school year and were given instructions for completing it in written form; they were asked to return it to the teacher within a week.

### Study variables

We collected sociodemographic data such as age and socioeconomic status, measured by the FAS scale. FAS is an individual socioeconomic index that reflects the affluence of a family by asking about the number of holidays per year, the number of computers and cars at home and the availability of a private bedroom. Subjects are classified in categories of low, middle and high socioeconomic status [18].

The variables obtained from the two FFQ were the children’s normal food intake frequency in relation to consumption in the last month, reported by the children and parents. Furthermore, after pooling data from the three administrations of YANA-C, the program provided information on the average number of portions eaten in a week. Some YANA-C food items were combined to homogenize the food groups analysed in all the questionnaires.

We created the descriptive variable, ‘Compliance with food recommendations’, from POIBA-HDWE according to the Spanish food recommendations [19], and generated a category of the same name. The remaining categories were grouped together as ‘Non-compliance with food recommendations’ but, since some recommendations were given for a food group instead of for individual foods, we combined rice, pasta, bread and grains as ‘carbohydrates’, and vegetables and fruits as another group.

We created a discrete variable, ‘Weekly food intake’, for POIBA-HDWE and POIBA-HDOCE to obtain data comparable with the gold standard, YANA-C. For both questionnaires, we designed variables by assigning a frequency to each food consumption category, using the week as a reference. ‘Never or <1 a month’ in the FFQ was considered 0 times per week in the new variable, ‘1–3 times/month’ was 0.5 times per week, ‘1–3 times/week’ was twice/week, ‘4–6 times/week’ was 5 times/week, ‘once/day’ was 7 times/week, ‘twice/day’ was 14 times/week, ‘≥3 times/day’ was 28 times/week, ‘once/week’, ‘twice/week’ and ‘≥3 times/week’ remained the same, and ‘1–6 times/week’ was 3.5 times/week. Since the three questionnaires differed in the way they enquired about grains, pasta, rice, bread and potatoes, we created different variables by pooling together food categories to make the variables comparable among the questionnaires.

A categorical variable, ‘weekly food intake’, was created for POIBA-HDWE and YANA-C, according to weekly consumption. For grains, bread, potatoes, fried potatoes, pasta, rice, soft drinks, chips, milk-based desserts, and pastries, we created the categories ‘<once/week’, ‘1–6 times/week’ and ‘≥once/day’. For vegetables, fruits, juices, sweets and dairy products, the categories ‘<once/week’, ‘1–3 times/week’ and ‘≥4 times/week’. Lastly, for meat, fish, legumes, eggs, nuts, and cold meats, we created the categories ‘<1/week’, ‘1–2/week’ and ‘≥3 times/week’.

We designed the ‘food consumers’ variable by dichotomising the frequencies from YANA-C and POIBA-HDWE, and assigning individuals as ‘non-consumers’ or ‘consumers’. ‘Non-consumers’ were those who reported that they eat POIBA-HDWE food items ‘never’, ‘less than once/month’ or ‘1–3 times/month’, and YANA-C food items ‘0 times/week’. The ‘consumer category’ included children who reported that they eat POIBA-HDWE or in YANA-C food items ‘once/week’.

Finally, weight and height were used to calculate the children’s body mass index (BMI). Categories were defined according to the World Health Organisation Z-score classification, which takes sex and age cut-off points into consideration [20]. The categories obtained were underweight, normal weight, overweight and obese; we re-categorized these as: excess weight (pooling overweight and obesity together) and normal weight (which included underweight due to the reduced number of cases).

### Statistical analysis

We conducted a descriptive analysis, stratified by sex, to determine the general characteristics of the sample. ‘Compliance with food recommendations’ was stratified by ‘excess weight’ and the Chi-square test or Fisher’s test were applied. We expected to find a low prevalence of compliance with food recommendations, especially for children with excess weight [8].

We conducted correlation analyses between POIBA-HDWE and POIBA-HDOCE and between POIBA-HDWE and YANA-C using the Spearman test for non-parametric variables. We expected stronger correlations for sweets, soft drinks, fried potatoes, chips, and meat, and weaker correlations for fish, dairy products, nuts, vegetables and fruits.

We determined the construct and criterion validity of the POIBA-HDWE FFQ using YANA-C as the gold standard and applied the Spearman test for non-parametric variables. We calculated the mean intake for each food item in POIBA-HDWE and YANA-C by using the discrete variable ‘weekly food intake’ and by applying a Wilcoxon test to compare means. Finally, we calculated the percentage of agreement and gross misclassification between the two food registers using the categorical variable ‘weekly food consumption’.

We analysed the internal consistency and reliability of POIBA-HDWE FFQ for the food frequency questions using Cronbach’s alpha, which was expected to be 0.6–0.7 [21,22]. Additionally, the validity of the POIBA-HDWE FFQ was evaluated by analysing the sensitivity, specificity, positive predictive values (PPV) and negative predictive values (NPV) with the variable ‘Food consumers’.

Data were analysed using the STATA statistics/data analysis version 11.2 statistical software package.

### Ethical issues

The POIBA study was approved by the ‘Parc de Salut Mar’ Clinical Research Ethics Committee (CEIC-Parc Salut Mar, Barcelona), and the participants’ families gave informed consent for their children to participate. Information was protected in accordance with the 1964 Declaration of Helsinki and amendments, and we observed the Spanish deontological codes and the Spanish data confidentiality law (Ley Orgànica 15/1999 de 13 de Diciembre de Protección de Datos de carácter personal [LOPD]).

## Results

The participation rate for the POIBA-HDWE FFQ was 96.8%. A total of 68.9% completed the entire 24-h recall process, and 88.5% participated in the correlation with POIBA-HDOCE (Figure 1). The mean age of the sample was 9.4 years and 61.9% of participants were boys. We found no significant differences in age or socioeconomic status between boys and girls. The percentage of excess weight was higher in boys (41.7%) than girls (25.0%).

The prevalence of consumption of the recommended portions was lower than 50.0% for all food groups except for the group of pasta, bread, grains and rice (84.2% for participants with normal weight and 65.0% for those with excess weight (Table 1). We found no differences in compliance with food recommendations between participants with excess weight and normal weight, except for dairy products (5.3% and 30.0%, respectively).

**Table 1.** Compliance with food recommendations in children stratified by excess weight (POIBA-HDWE FFQ).

	Normal weight^a^ n(%) = 39 (65.0)		Excess weight^a^ n (%) = 21 (35.0)	
	Recommended	Not recommended	Recommended	Not recommended
**Bread, pasta, grains and rice** (4–6 portions/day)	32 (84.2)	6 (15.8)	13 (65.0)	7 (35.0)
**Fruits and vegetables** (5 or more portions/day)	4 (10.5)	34 (89.5)	2 (10.0)	18 (90.0)
**Dairy** (3 portions/day)	**2 (5.3)^b^**	36 (94.7)	**6 (30.0)^b^**	14 (70)
**Meat** (3–4 portions/week)	12 (31.6)	26 (68.4)	5 (25.0)	15 (75.0)
**Fish** (3–4 portions/week)	5 (13.2)	33 (86.8)	4 (20.0)	16 (80.0)
**Eggs** (3–4 portions/week)	3 (7.9)	35 (92.1)	0	20 (100)
**Legumes** (3–4 portions/week)	3 (7.9)	35 (92.1)	0	20 (100)
**Nuts** (3–7 portions/week)	7 (18.4)	31 (81.6)	1 (5.0)	19 (95.0)
**Sweets, pastries, snacks (**occasionally)	8 (21.1)	30 (79.0)	7 (35.0)	13 (65.0)

^a^The IMC could not be obtained for three out of 61 students who completed the FFQ.

^b^Significant differences were found in dairy consumption within excess weight and normal weight (Chi-square test at 0.05 significance).

POIBA- HDOCE: POIBA-How do our children Food Frequency Questionnaire.

POIBA-HDWE and POIBA-HDOCE showed moderate correlation for soft drinks (r = 0.49; *p* < 0.01), nuts (r = 0.59; *p* < 0.01), dairy products (r = 0.41; *p* < 0.01) and juices (r = 0.49; *p* < 0.01) (Table 2). Finally, we found moderate correlation between YANA-C and POIBA-HDOCE for nuts (r = 0.44; *p* < 0.01), fruits (r = 0.43; *p* < 0.01) and juices (r = 0.62; *p* < 0.01).

**Table 2.** Spearman Correlations between “POIBA- HDOCE, POIBA-HDWE FFQ and YANA-C (gold standard).

	POIBA-HDOCE Vs POIBA-HDWE		POIBA- HDOCE Vs YANA-C	
Food	r^a^ coefficient	p value	r^a^ coefficient	p value
Pastries	−0.036	0.798	0.251	0.114
Chips	0.133	0.335	0.254	0.109
Bread	0.242	0.078	0.159	0.321
Fried Potatoes	0.089	0.523	–	–
Milk-based desserts	0.036	0.792	0.010	0.910
Soft drinks	**0.485**	<0.001	**0.420**	0.005
Meat	0.225	0.108	−0.160	0.320
Sweets	0.310	0.023	0.255	0.107
Nuts	**0.590**	<0.001	**0.440**	0.005
Legumes	0.200	0.149	0.279	0.086
Eggs	−0.004	0.976	−0.008	0.959
Fish	0.221	0.115	0.350	0.025
Dairy	**0.410**	0.002	0.238	0.135
Fruits	0.250	0.064	**0.431**	0.004
Juices	**0.490**	<0.001	**0.620**	<0.001
Vegetables	−0.020	0.885	−0.060	0.690
Cold meats	0.220	0.115	−0.090	0.570
Grains, rice and pasta	–	–	0.101	0.520
Fried and boiled Potatoes	–	–	0.236	0.137

^a^Spearman correlation (ρ).

POIBA- HDOCE: POIBA-How do our children eat Food Frequency Questionnaire.

POIBA-HDWE: POIBA- How do we eat Food Frequency Questionnaire.

Table 3 shows the relative validation of POIBA-HDWE FFQ compared with the gold standard YANA-C. We found moderate correlations for fried potatoes (r = 0.42; *p* = 0.01), dairy products (r = 0.53; *p* < 0.01), juices (r = 0.41; *p* < 0.01), and grains (r = 0.50; *p* < 0.01). There were significant differences between POIBA-HDWE FFQ and the gold standard in terms of mean consumption frequency, except for pastries (*p* = 0.53), pasta (*p* = 0.81) and cold meats (*p* = 0.63). This indicates that POIBA-HDWE overestimates the consumption of most foods except meat (ratio = 0.5, *p* < 0.01) and sweets (ratio = 0.71, *p* < 0.01), which were underestimated. The percentage of agreement varied from 23.8% to 83.3%, while gross misclassification ranged from 0 to 59.5%.

**Table 3.** Relative validity of POIBA-HDWE FFQ compared to YANA-C (gold standard).

		Mean^b^ (SE) consumption frequency					
Variable	r^a^	Yana-C	POIBA-HDWE	P-value^c^	Ratio^d^ POIBA/Yana-C	% agreement^e^	% gross misclassification^e^
Boiled or baked potatoes	−0.040	0.44 (0.10)	3.95 (0.59)	<0.01	9.76	40.48	14.29
Pastries	0.042	2.37 (0.33)	3.11 (0.51)	0.53	1.31	35.71	2.38
Legumes	0.071	0.12 (0.06)	1.33 (0.13)	<0.01	11.20	83.33	7.14
Rice	0.107	0.56 (0.12)	2.49 (0.22)	<0.01	4.35	52.38	0.00
Pasta	0.115	3.16 (0.18)	3.42 (0.36)	0.81	1.10	73.81	0.00
Vegetables	0.129	1.98 (0.27)	5.90 (0.87)	<0.01	2.99	33.33	11.90
Chips	0.162	0.28 (0.10)	1.79 (0.39)	<0.01	6.25	57.14	0.00
Bread	0.164	3.81 (0.32)	8.51 (0.72)	<0.01	2.23	30.95	0.00
Cold meats	−0.176	1.77 (0.26)	2.35 (0.48)	0.63	1.35	23.81	35.71
Eggs	0.182	0.63 (0.12)	1.29 (0.12)	<0.01	2.08	54.76	0.00
Meat	−0.195	3.70 (0.25)	1.85 (0.14)	<0.01	0.50	23.81	59.52
Milk-based desserts	−0.211	0.91 (0.12)	3.69 (0.61)	<0.01	4.31	38.10	7.14
Fish	0.212	0.67 (0.14)	1.50 (0.12)	<0.01	2.17	52.38	9.52
Nuts	0.310	0.19 (0.08)	1.14 (0.16)	<0.01	6.00	73.81	11.90
Fruits	0.323	4.05 (0.28)	12.21 (1.33)	<0.01	3.05	59.52	2.38
Sweets	0.324	1.95 (0.34)	0.71 (0.11)	0.01	0.37	50.00	9.52
Grains, rice and pasta	0.339	8.50 (0.41)	10.73 (0.77)	<0.01	0.57	38.10	0.00
Soft drinks	0.357	0.51 (0.11)	3.40 (0.58)	<0.01	6.81	52.38	9.52
Juices	**0.407**	1.35 (0.22)	5.77 (0.99)	<0.01	4.18	38.10	14.29
Fried potatoes	**0.416**	0.56 (0.12)	1.69 (0.23)	<0.01	2.96	73.81	0.00
Grains	**0.498**	1.02 (0.19)	4.82 (0.53)	<0.01	4.73	33.33	11.90
Dairy	**0.531**	6.12 (0.43)	11.55 (1.32)	<0.01	1.92	78.57	0.00

^a^Spearman correlation (ρ).

^b^POIBA-HDWE and YANA-C mean (Standard error, SE) consumption frequency.

^c^Wilcoxon test for mean comparison.

^d^Ratio between POIBA-HDWE and YANA-C.

^e^Percentage of agreement and percentage of gross misclassification between categories in POIBA-HDWE and YANA-C.

POIBA-HDWE: POIBA- How do we eat Food Frequency Questionnaire.

Food frequency questions showed a homogeneity of 0.69, and a sensitivity of over 60% between POIBA-HDWE and YANA-C for all food items except for chips (37.5%) and sweets (51.7%). Specificity was mostly below 60% except for pasta (100%), fried potatoes (79.2%), sweets (76.9%) and chips (67.6%) (Table 4).

**Table 4.** Sensitivity and specificity of the POIBA-HDWE FFQ in detecting children’s consumption.

	Sensitivity	Specificity	PPV	NPV
Bread	–	–	–	–
Rice	**70.0**	36.4	50.0	57.1
Pasta	**82.9**	**100.0**	100.0	12.5
Grains	**95.5**	45.0	65.6	90.0
Vegetables	**90.9**	11.1	78.9	25.0
Fruits	**97.6**	0.0	97.6	0.0
Potatoes	**73.3**	29.6	36.7	66.7
Potatoes fried	66.7	**79.2**	70.6	76.0
Meat	**82.9**	0.0	97.1	0.0
Fish	**94.7**	21.7	50.0	83.3
Legumes	**100.0**	23.7	12.1	100.0
Eggs	63.2	21.7	40.0	41.7
Nuts	**100.0**	41.7	22.2	100
Dairy	–	–	–	–
Juices	**96.0**	47.1	72.7	88.9
Sweets	51.7	**76.9**	83.3	41.7
Cold meats	**82.8**	15.4	68.6	28.6
Pastries	60.0	42.9	84.0	17.6
Chips	37.5	67.6	21.4	82.1
Milk-based desserts	60.7	28.6	63.0	26.7
Soft drinks	**76.5**	56.0	54.2	77.8

PPV: Positive Predictive Value.

NPV: Negative Predictive Value.

POIBA-HDWE: POIBA- How do we eat Food Frequency Questionnaire.

## Discussion

The POIBA-HDWE food frequency questionnaire obtained moderate correlations with the gold standard with a high percentage of agreement and consistency.

We found moderate correlations between POIBA-HDOCE and YANA-C for soft drinks, nuts, fruits and juices, and similar correlations between the parent’s FFQ and the children’s FFQ for the aforementioned items, excluding fruits. These results were in line with our initial hypothesis only for soft drinks and fried potatoes. At these ages, parents do not have complete control over what their children eat, since most have lunch at school and parents generally do not verify what their children have eaten [4]. Unsurprisingly, the items with the strongest correlation were those not commonly consumed at school, such as soft drinks and juices, and those that parents give special attention to, such as fruits and nuts. The POIBA-HDWE FFQ and YANA-C showed moderate correlations, even among nine-year-old pupils [23,24], for fried potatoes, soft drinks, dairy products and juices, which is consistent with previous reports [25]. At these ages, children remember items better according to their preference, which could be the case for dairy products, juices, fried potatoes or soft drinks [4].

In contrast, the items with the weakest correlations between the family questionnaire and YANA-C or the children’s questionnaire were vegetables, eggs, meat and cold meat. This could be attributed to parents not always knowing what their children have eaten at school and, moreover, the parents’ weight can influence their reports about their children’s food consumption. Finally, children sometimes do not know how food was cooked or which ingredients it contained [4]. The items with the worse correlations between the POIBA-HDWE FFQ and YANA-C were potatoes, legumes, pasta, rice and bread. Children can omit or forget some items they have eaten, such as side dishes (although they are commonly eaten) or rare food groups, as these showed the greatest mismatch between respondent and observer reports [23]. Other authors have also reported that responses on portions and servings may differ between FFQ and recall and that this mismatch could influence correlations [26]. Other reasons cited were related to the difficulty of 24-h recalls in capturing infrequently consumed products [27,28].

In relation to mean consumption, the POIBA-HDWE FFQ overestimated some types of food. While the questionnaire does not accurately estimate the amount of food consumed, it is completely valid for classifying children because the percentages of agreement are high and the percentages of gross misclassification are low. Similar results were found by Vereecken et al. [29].

The POIBA-HDWE FFQ had a sensitivity of over 60% for most food items, except for sweets and chips. Furthermore, PPV was generally good for most items, indicating that when the FFQ detects a consumer, he/she is normally correctly classified. The questionnaire designed seems to be a good tool for detecting those children who truly consume the food items studied. However, overall specificity was low except for pasta, fried potatoes, sweets and chips, and half of the items showed high NPV. The FFQ did not accurately detect non-consumers for most of the items, but the children classified as non-consumers were probably not misclassified [23,28].

Eating habits are a major factor for overweight and obesity, and we observed that children’s compliance with the food recommendations was low for all food groups except grains, pasta, bread and rice. It is reported that food consumption is shifting towards a more processed diet [30]; therefore it is especially important to count on validated tools, such as POIBA_HDWE, to monitor the food intake of children.

This study had some limitations, one of which was that it was based on a convenience sample that did not include children from the lowest socioeconomic level. However, the objective was to validate the questionnaire and the available sample was suitable for this aim. Since children from lower socioeconomic levels tend to omit more food items than children from other socioeconomic strata, special care should be taken when applying questionnaires in these socioeconomic levels, although we do not expect a large bias [31]. Furthermore, the sample consisted of only 42 students, which could limit the results of our study. We found moderate correlations, but we believe that more and stronger correlations could have been found with a larger sample. Another limitation is that YANA-C is a validated tool for children over 11 years old, although we administered it to children between 8 and 11 years old and took precautions to minimize the discrepancies. During the administration process, a professional guided the children. In addition, various studies show that children over seven years old are able to complete food records in the format of 24-h recall [23,30]. We were unable to measure the reliability of the FFQ for feasibility reasons, and, finally, some bias could have been introduced during the analysis because the recall time differs between the two methods used (the POIBA-HDWE FFQ asks about normal food intake in the last month, while the YANA-C asks about the day before). Nevertheless, other studies have shown good results using the same recording tools [28].

The study also had some strengths. First, we conducted a pilot administration to ensure that the children understood the questionnaire and that the study design was well recommended. Secondly, FFQ for schoolchildren are highly limited, and non-existent in Barcelona, and this study presents a cost-affordable validated tool that can be used for other studies in a similar context. We agree with other authors in believing that POIBA-HDWE, as a FFQ, is a good tool to classify consumers and non-consumers [23,24]. In addition, in future studies, it will allow for comparison of different foods in the same person over time.

Regarding participants’ age, Lillegaard and Hunsberger also studied an FFQ in 9– 10-year-old schoolchildren and, as in our study, confirmed that they are useful in participants younger than 10 years old [23,27]. Lillegaard found that the results obtained in nine-year-old students were even better than those obtained in participants aged 13 years [23].

## Conclusion

The POIBA-HDWE FFQ shows moderate correlation and sensitivity, making it an easy and affordable tool for recording food frequency in children under 10 years of age. In future studies, we recommend using larger samples and representation of all socioeconomic levels.
